# Etiology, Pathogenicity, and Fungicide Management of Pigeonpea Shoot Dieback Caused by *Lasiodiplodia theobromae* in China

**DOI:** 10.3390/jof12070474

**Published:** 2026-06-28

**Authors:** Feiyun Huang, Xi Xu, Yanzhong Li

**Affiliations:** 1 State Key Laboratory of Herbage Improvement and Grassland Agro-Ecosystems, College of Pastoral Agriculture Science and Technology, Lanzhou University, Lanzhou 730020, China; 2 Engineering Research Center of Grassland Industry, Ministry of Education, Lanzhou 730020, China

**Keywords:** pigeonpea, *Cajanus cajan*, *Lasiodiplodia theobromae*, shoot tip dieback, pathogen identification, host growth suppression, fungicide efficacy

## Abstract

Pigeonpea (*Cajanus cajan*) shoot tip dieback was recently observed in Danzhou, Hainan Province, China, with disease incidence of 30–100% in affected fields, but its causal agent and management options remained unclear. This study aimed to isolate and identify the fungal agent associated with the disease, verify its pathogenicity, characterize its biological traits and effects on host growth, and screen candidate fungicides. Fungal isolates were obtained from symptomatic shoot margins and purified by hyphal-tip and single-spore isolation. Representative isolates LYZ0717, LYZ0718, and LYZ0719 were characterized by colony morphology, pycnidial and conidial traits, and combined phylogenetic analysis of ITS and *TEF1-α* sequences. These analyses identified the isolates as *Lasiodiplodia theobromae*. Spray inoculation of 20-day-old pigeonpea seedlings reproduced typical shoot tip dieback symptoms within 6 days, and the inoculated fungus was reisolated from symptomatic tissues, fulfilling Koch’s postulates. The pathogen grew over a pH range of 3–11 and at temperatures of 5–35 °C, with optimal mycelial growth at 25–30 °C and maximum sporulation under acidic conditions. At 20 days after inoculation, infection reduced plant height, dry weight, and total flavonoid content by 45.21%, 75.88%, and 42.19%, respectively. Among the seven fungicides tested, trifloxystrobin–tebuconazole and thiophanate-methyl showed the strongest in vitro inhibition, with EC_50_ values of 0.0270 and 0.0614 ppm, respectively. In greenhouse pot experiments, thiophanate-methyl achieved the highest disease control efficacy, reaching 64.35% after the second application. These findings demonstrate that *L. theobromae* is the causal agent of pigeonpea shoot tip dieback in China and provide a basis for disease diagnosis, biological understanding, and fungicide-based management.

## 1. Introduction

Pigeonpea (*Cajanus cajan* L.) is an important legume crop and a major source of dietary protein in many regions of Asia and Africa, where it contributes substantially to food and nutritional security [1]. In China, pigeonpea is cultivated mainly in tropical and subtropical regions and is valued for food, fodder, and medicinal applications [2]. However, its production remains constrained by multiple biotic stresses, particularly fungal diseases [3,4,5]. More than 50 pathogens have been reported to infect pigeonpea worldwide [6].

Among them, Fusarium wilt, caused by *Fusarium udum*, and Phytophthora blight, caused by *Phytophthora drechsleri* f. sp. *cajani* or *Phytophthora cajani*, are among the most destructive diseases, severely reducing yield and quality while threatening farmers’ income and food security [7,8,9,10,11]. In India, where these soil-borne pathogens are widespread across pigeonpea-growing areas, grain yield losses can reach 30–100% [12]. Fusarium wilt alone causes annual economic losses of approximately US$113 million in Indian pigeonpea production [13]. These losses underscore the vulnerability of pigeonpea production systems to destructive fungal pathogens and the need for rapid recognition of emerging disease threats.

*L. theobromae* is a broad-host-range fungal pathogen capable of infecting over 500 plant species, including mango (*Mangifera indica*), grape (*Vitis vinifera*), blueberry (*Vaccinium corymbosum*), rice (*Oryza sativa*), and tea (*Camellia sinensis*), thereby posing a severe threat to global agricultural and forestry production [14]. Because this pathogen occurs on diverse hosts, induces a wide range of symptoms, and is frequently associated with other closely related Botryosphaeriaceae fungi, accurate identification is essential for disease diagnosis, epidemiological surveillance, and effective disease management [15,16,17]. Morphological characterization remains an important first step for the preliminary identification of *L. theobromae* and related Botryosphaeriaceae fungi. Colonies of *L. theobromae* are generally fast-growing and initially white to grayish, later becoming dark gray, olivaceous, or nearly black with age [15,17]. The fungus produces dark pycnidial conidiomata, either singly or in clusters, on infected host tissues or in culture [15,18]. Conidia are produced from hyaline conidiogenous cells and undergo a characteristic developmental transition: immature conidia are hyaline, aseptate, thick-walled, and ellipsoid to ovoid, whereas mature conidia become brown to dark brown, usually one-septate, and longitudinally striate [15,17]. The presence of pycnidial paraphyses and longitudinal striations on mature conidia provides useful diagnostic evidence for the genus *Lasiodiplodia* [18]. However, morphology alone is insufficient for reliable species delimitation because colony appearance, conidial dimensions, pigmentation, septation, and striation patterns often overlap among closely related species, particularly *L. theobromae* and *L. pseudotheobromae*. Therefore, morphological observations should be integrated with multilocus phylogenetic analyses, especially using ITS and protein-coding loci such as *TEF1-α*, to ensure accurate species identification [19].

Across diverse hosts, this pathogen causes dieback, gummosis, leaf spot, branch dieback, and fruit rot, although disease expression and aggressiveness vary markedly among fungal strains and host species [20,21,22,23]. Its predominance in tropical and subtropical regions is of particular concern because recent warming trends may have facilitated the expansion of its geographic distribution and host range over the past two decades [24,25,26]. Moreover, *L. theobromae* can persist asymptomatically as an endophyte and switch to a pathogenic lifestyle under favorable environmental conditions or host stress, a dual strategy that complicates disease detection, epidemiological surveillance, and management [26]. Consequently, effective disease suppression requires an integrated management strategy that combines the use of healthy planting material, sanitation through the removal of infected tissues, minimization of wounds that may serve as infection courts, and the judicious application of chemical or biological control agents. Several fungicides have been evaluated against *L. theobromae* in different host–pathogen systems [27,28,29,30]. In sapote mamey, fungicide applications reduced dieback incidence in grafted plants, whereas in vitro assays showed strong inhibition of fungal growth by cyprodinil + fludioxonil, pyraclostrobin + boscalid, prochloraz, tebuconazole, iprodione, and thiabendazole [27]. Similarly, tebuconazole, pyraclostrobin, mancozeb, fosetyl-Al, and azoxystrobin, together with several biological control agents, have been assessed for protecting grapevine pruning wounds against infection by *L. theobromae* [28,31]. However, fungicide efficacy may vary depending on host species, pathogen isolate, application timing, and environmental conditions, and strong in vitro activity does not necessarily translate into effective disease suppression in planta [27,28,32]. Moreover, repeated use of site-specific fungicides, particularly benzimidazoles, demethylation inhibitors, and quinone outside inhibitors, may increase the risk of resistance development, highlighting the need for rotation with, or mixtures of, compounds with different modes of action [29,30,33]. Therefore, evaluating candidate fungicides under both in vitro and plant-based conditions is essential for identifying effective and practical options for managing pigeonpea shoot tip dieback caused by *L. theobromae*.

Previous studies have reported an association between *L. theobromae* and *C. cajan* in other regions; however, confirmed etiological evidence for pigeonpea shoot tip dieback caused by this pathogen in China is lacking. Moreover, the biological characteristics of the pathogen population associated with pigeonpea in China and effective chemical options for disease suppression remain unclear. Therefore, the objectives of this study were to: (i) isolate and identify the causal agent associated with pigeonpea shoot tip dieback in Danzhou, Hainan Province, using morphological and molecular phylogenetic approaches; (ii) confirm pathogenicity and characterize key biological traits affecting mycelial growth and sporulation; and (iii) evaluate candidate fungicides using in vitro sensitivity assays and greenhouse pot experiments.

## 2. Materials and Methods

### 2.1. Field Surveys and Sample Collection

Systematic field surveys were conducted in February, May, and August 2023 at the Pigeonpea (*Cajanus cajan*) Breeding Base of the Tropical Crops Genetic Resources Institute, Chinese Academy of Tropical Agricultural Sciences (CATAS), Danzhou, Hainan Province, China (19°11′–19°55′ N, 108°56′–109°45′ E; elevation, 168.7 m). The study site has a tropical monsoon climate, with a mean annual temperature of 23.3 °C and annual precipitation of 1800–2000 mm. The base mainly propagates six pigeonpea germplasm accessions: D23408, D23442, D23445, D23452, D23458, and D23524. During the surveys, pigeonpea plants at different developmental stages were examined for dieback symptoms. Typical symptoms were characterized by necrosis of the apical shoots, which subsequently progressed basipetally into the main stem and lateral branches. In severely affected plants, the aboveground tissues died, whereas the root system remained viable. Symptomatic branches were collected, placed in sterile paper bags, and transported to the laboratory for pathogen isolation. Six fields were surveyed; five quadrats per field; 20 plants per quadrat. Disease incidence was estimated using a random quadrat sampling method and calculated as follows:

$$I\;\left(\%\right)=\left(n/N\right)\times 100$$

where *n* represents the number of infected plants and *N* represents the total number of plants surveyed.

### 2.2. Pathogen Isolation and Purification

Symptomatic shoots were rinsed under running tap water and blotted dry. Tissue segments approximately 3–5 mm in length were excised from the interface between healthy and necrotic tissues, surface-disinfested in 75% ethanol for 30 s, followed by 1% sodium hypochlorite for 60 s, rinsed three times with sterile distilled water, and dried on sterile filter paper. The disinfected segments were placed on potato dextrose agar (PDA) supplemented with chloramphenicol (100 mg/L) and incubated at 28 °C in darkness. Emerging hyphal tips were transferred to fresh PDA plates to obtain pure cultures. Single-spore isolates were generated by spreading conidial suspensions onto water agar and incubating them at 28 °C in darkness, followed by transferring germinated conidia to PDA. Representative isolates were maintained on PDA slants at 4 °C and preserved as glycerol stocks at −80 °C. A total of 86 fungal isolates were obtained from 90 symptomatic shoot tissue segments. Based on preliminary colony characteristics on PDA, including rapid mycelial growth, initially white floccose colonies that gradually became gray to dark gray or black, and pycnidial development, 76 isolates were assigned to the dominant morphotype consistent with *Lasiodiplodia* spp. Three single-spore isolates, LYZ0717, LYZ0718, and LYZ0719, were selected for subsequent morphological, molecular, and pathogenicity analyses because they represented the dominant morphotype, showed stable colony characteristics, and produced sufficient pycnidia and conidia for morphological observation and inoculum preparation.

### 2.3. Morphological Characterization

For morphological characterization, the representative isolates LYZ0717, LYZ0718, and LYZ0719 were cultured on PDA at 28 °C in darkness. Colony color, texture, growth rate, and pycnidial development were recorded daily. To induce sporulation, cultures were incubated at 28 °C under a 12 h light/12 h dark photoperiod for 12 days, after which pycnidia and conidia were observed. Pycnidia and conidia were examined using a light microscope (Olympus CX33; Olympus Corporation, Hachioji, Tokyo, Japan). For each isolate, at least 50 conidia were measured for length and width, and representative photomicrographs were captured.

### 2.4. Genomic DNA Extraction and Multi-Locus Phylogenetic Analysis

Genomic DNA was extracted from the mycelia of isolates LYZ0717, LYZ0718, and LYZ0719. The isolates were grown on potato dextrose agar (PDA) at 28 °C in darkness for 7 days. Approximately 100–120 mg of fresh mycelium was harvested from each isolate and homogenized using a Retsch mixer (MM 400; Retsch GmbH, Haan, Germany) mill equipped with sterile steel beads. Genomic DNA was extracted with the HP Fungal DNA Kit (D3195, Omega Bio-tek, Norcross, GA, USA) according to the manufacturer’s instructions. The internal transcribed spacer (ITS) region and the translation elongation factor 1-alpha (*TEF1-α*) gene were amplified by PCR using primer pairs ITS1 (TCCGTAGGTGAACCTGCGG)/ITS4 (TCCTCCGCTTATTGATATGC) and EF1-688F (CGGTCACTTGATCTACAAGTGC)/EF1-1251R (CCTCGAACTCACCAGTACCG), respectively. Amplification was performed on a BIOER Gene Explorer thermal cycler. PCR products were sequenced by Sangon Biotech (Shanghai, China). Raw sequences were assembled and trimmed using SeqMan and deposited in GenBank under the following accession numbers: ITS, PV111792-PV111794; *TEF1-α*, PV125493-PV125495. Phylogenetic analyses were conducted in MEGA 11. Reference sequences of *Lasiodiplodia* and related Botryosphaeriaceae species were retrieved from GenBank. A neighbor-joining tree was constructed from the concatenated ITS + *TEF1-α* dataset with 1000 bootstrap replicates to infer the taxonomic placement of the isolates.

### 2.5. Pathogenicity Assays

Pathogenicity assays were conducted using healthy 20-day-old pigeonpea seedlings of germplasm D22006. Seeds were surface-disinfested with 75% ethanol for 30 s, followed by 1% sodium hypochlorite for 5 min, rinsed three times with sterile distilled water, and sown in a sterilized mixture of sandy loam soil and organic nutrient soil (2:1, *v*/*v*) in plastic pots measuring 15 cm in diameter and 12 cm in height. Seedlings were maintained under greenhouse conditions at 25 ± 2 °C, 70–85% relative humidity, and a 16 h light/8 h dark photoperiod. Conidial suspensions were prepared from 30-day-old sporulating cultures of each tested isolate, namely LYZ0717, LYZ0718, and LYZ0719. The suspensions were filtered through sterile gauze and adjusted to 1 × 10^6^ conidia mL^−1^ using a hemocytometer. For each treatment group, five pots of healthy and uniform seedlings were used for inoculation, with four plants per pot. Each pot was sprayed with 15 mL of conidial suspension until the plant surfaces were uniformly moistened. Control plants were sprayed with an equal volume of sterile distilled water. After inoculation, plants were covered with black plastic bags for 48 h to maintain high humidity and then maintained under greenhouse conditions.

Disease severity was evaluated using a 0–4 scale modified according to the percentage of necrotic aboveground tissue [34]: 0 = no visible symptoms; 1 = slight shoot-tip wilting or necrosis affecting less than 25% of the aboveground tissues; 2 = necrosis affecting 25–50% of the aboveground tissues; 3 = severe dieback affecting 50–75% of the aboveground tissues with leaf abscission; and 4 = more than 75% necrosis of the aboveground tissues or plant death. The disease index (DI) was calculated as follows:

$$DI(\%)=\frac{\sum_{i=0}^{4}\left(n_{i}\times i\right)}{N\times 4}\times 100$$

where *DI* represents the disease index; *i* represents the disease class value ranging from 0 to 4; *n*_*i*_ represents the number of plants in disease class *i*; *N* represents the total number of plants assessed; and 4 represents the highest disease class value.

For re-isolation, small tissue segments were excised from the margins of symptomatic lesions and isolated and cultured as described in Section 2.2. The re-isolated fungi were compared with the original inoculated isolates based on colony morphology and conidial characteristics, and representative re-isolates were further confirmed by ITS and/or *TEF1-α* sequencing.

### 2.6. Assessment of Biological Characteristics

The in vitro biological characteristics of the representative pathogenic isolates LYZ0717, LYZ0718, and LYZ0719 were evaluated. Unless otherwise stated, all assays included five replicates and were independently repeated three times.

Culture media: Mycelial growth and sporulation were evaluated on seven culture media: potato dextrose agar (PDA), oatmeal agar (OMA), corn meal agar (CMA), potato sucrose agar (PSA), potato carrot agar (PCA), wheat straw decoction agar (WHDA), and 2% water agar (WA) [35].

pH: The effect of pH on growth and sporulation was tested on PDA adjusted to pH levels ranging from 3 to 11 (at intervals of 1) using 1 mol L^−1^ NaOH and 1 mol L ^−1^ HCl.

Temperature: Growth assays were conducted on PDA at seven distinct temperatures: 5, 10, 15, 20, 25, 30, and 35 °C.

UV Irradiation: To determine the effect of UV radiation on mycelial growth and sporulation, mycelial plugs were transferred to fresh PDA plates and incubated at 25 °C in the dark for 2 days. Cultures were then exposed to UV light for 0, 30, 60, 90, or 120 min. Following exposure, all plates were incubated in the dark at 25 °C.

Photoperiod: The influence of light was assessed under three conditions: 24 h continuous dark, 24 h continuous light, and a 12 h light/12 h dark cycle.

### 2.7. Impact of L. theobromae on Pigeonpea Growth

To assess the effects of the pathogen on host growth and development, the confirmed pathogenic isolates *L. theobromae* LYZ0717, LYZ0718, and LYZ0719 were used to inoculate healthy 20-day-old pigeonpea seedlings approximately 15 cm in height. The tested pigeonpea germplasm was D22006. For each treatment group, five pots of healthy and uniform seedlings were used for inoculation, with four plants per pot. The inoculation procedure followed the protocol described in Section 2.5. Seedlings in the control treatment (CK) were mock-inoculated with an equivalent volume of sterile distilled water. At 20 days post-inoculation (dpi), plants were harvested for measurement of growth-related traits, including plant height (cm), root length (cm), stem diameter (mm), fresh weight (g), and dry weight (g). In addition, total flavonoid content in oven-dried plant tissues was quantified using a Total Flavonoid Assay Kit (Cat. No. G0118W; Suzhou Grace Biotechnology Co., Ltd., Suzhou, China) according to the manufacturer’s instructions.

### 2.8. Fungicide Sensitivity Assay

Seven commercial fungicides were selected for sensitivity testing based on their reported efficacy against wilt and dieback diseases and their distinct modes of action (Table 1). In vitro fungicide sensitivity was evaluated using a mycelial growth inhibition assay based on the poisoned food technique [36]. Preliminary assays were conducted using a concentration series of 0.2, 0.4, 0.8, 1.6, and 3.2 ppm to identify effective inhibitory ranges. Based on these preliminary results, a tailored concentration series was established for each fungicide. Fungicide stock solutions were incorporated into molten PDA at a ratio of 1:1000 (*v*/*v*) to prepare fungicide-amended media. Control plates (CK) were prepared by adding an equivalent volume of sterile distilled water to PDA. A 6 mm mycelial plug was placed at the center of each plate, and plates were incubated at 25 °C in darkness. After 7 days, colony diameter was measured along two perpendicular axes, and the mean value was used for analysis. The percentage inhibition of mycelial growth was calculated as follows:

$$Inhibition\;Rate\;\left(\%\right)=\frac{\left(D_{ck}-d\right)-{(D}_{tr}-d)}{D_{ck}-d}\times 100$$

where *D_ck_* is the mean colony diameter of the control, *D_tr_* is the mean colony diameter of the fungicide treatment, and *d* is the diameter of the inoculation plug (6 mm).

The median effective concentration required to inhibit mycelial growth by 50% (EC_50_) was estimated by linear regression of the probit-transformed inhibition rate (y) against the base-10 logarithm of fungicide concentration (x) [37]. Regression equations, correlation coefficients, and EC_50_ values were generated using IBM SPSS Statistics 27.0 (IBM Corp., Armonk, NY, USA). Each treatment was represented by five replicate plates, and the entire experiment was independently repeated three times.

**Table 1:** List of tested chemical fungicides and their specifications.

Fungicide	Active Ingredient Concentration	Formulation	Mode of Action/Activity	Manufacturer	Reference
Mancozeb-Carbendazim	60%	WP	Systemic/Protectant	Jinan Luba Pesticide Co., Ltd. (Shandong, China)	[38]
Azoxystrobin	250 g/L	SC	Systemic/Protectant/ Curative	Syngenta Nantong Crop Protection Co., Ltd. (Jiangsu, China)	[39]
Trifloxystrobin-tebuconazole	75%	WDG	Systemic/Protectant/ Curative	Bayer AG (Leverkusen, Germany)	[40]
Thiram-Ziram	40%	WP	Systemic/Protectant/ Curative	Hebei Zanfeng Bioengineering Co., Ltd. (Hengshui, China)	[41]
Chlorothalonil	75%	WP	Contact/Protectant	Bayer AG (Leverkusen, Germany)	[42]
Triadimefon	15%	WP	Systemic/Protectant/ Curative	Jiangsu Jianpai Agrochemical Co., Ltd. (Yancheng, China)	[43]
Thiophanate-methyl	70%	WP	Systemic/Protectant/ Curative	Zhejiang Weierda Chemical Co., Ltd. (Hangzhou, China)	[38]

### 2.9. Greenhouse Pot Experiments for Disease Control

Based on the results of the in vitro fungicide sensitivity assays, the two fungicides with the lowest EC_50_ values, 70% thiophanate-methyl and 75% trifloxystrobin–tebuconazole, were selected for further evaluation of their disease control efficacy under greenhouse conditions. Two sterile-water control treatments were included: a pathogen-inoculated control and a non-inoculated control. The pigeonpea accession D22006 was used in this experiment, and all seeds were surface-disinfested according to the procedure described in Section 2.5. Each treatment consisted of five pots, with four healthy seedlings of uniform growth per pot, resulting in a total of 20 seedlings per treatment. The pathogen-inoculated control received pathogen inoculation followed by sterile distilled water instead of fungicide, whereas the non-inoculated control received sterile distilled water without pathogen inoculation. Pathogen inoculation was performed following the procedure described in Section 2.5. Fungicides were applied as whole-plant sprays at a volume of 3–5 mL per plant when initial symptoms were observed, followed by a second application 10 days later. Disease incidence, disease index [36], and mortality rate were recorded before the first application, before the second application, and 10 days after the second application. Control efficacy was calculated as follows:

$$Control\;Efficacy\;(\%)=\frac{DI_{ck}-DI_{treatment}}{DI_{ck}}\times 100$$

where *DI_ck_* is the disease index of the positive control and *DI_treatment_* is the disease index of the fungicide treatment.

### 2.10. Data Analysis

Statistical analyses were conducted using SPSS version 27.0. Following one-way analysis of variance, mean comparisons among treatments were performed using Duncan’s multiple range test at *p* < 0.05. Data are presented as means ± standard error (SE). Figures were generated using Origin 2024 and refined in Adobe Illustrator 2024.

## 3. Results

### 3.1. Occurrence and Symptomatology of Pigeonpea Shoot Tip Dieback

**Figure 1:**
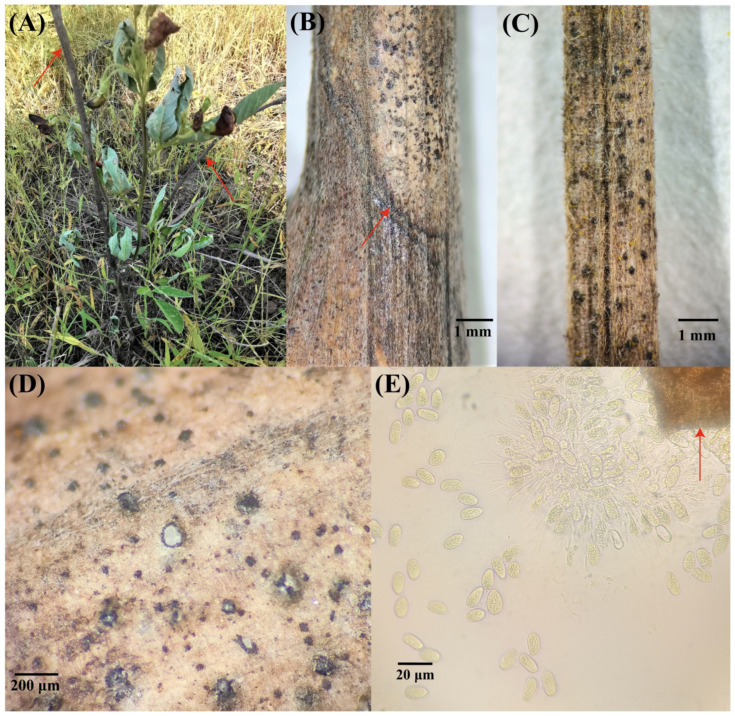
Symptoms of pigeonpea shoot tip dieback observed on pigeonpea (*Cajanus cajan*) plants in Danzhou, Hainan Province, China. (**A**): Incidence in field; (**B**,**C**): Close-up of stem symptoms of diseased plants; (**D**,**E**): Black pycnidia and conidia.

Field surveys showed that pigeonpea (*Cajanus cajan*) shoot tip dieback occurred at all surveyed planting sites, with disease incidence ranging from 30% to 100%. Early symptoms were characterized by wilting and necrosis of the apical shoots, which subsequently progressed basipetally to lower branches and lateral shoots (Figure 1A). At advanced stages of disease development, a clear demarcation developed between necrotic and healthy tissues (Figure 1B). Numerous black, granular structures were observed on the lesion surface and were identified as pycnidia (Figure 1C). When sectioned, these pycnidia appeared white internally (Figure 1D) and contained hyaline, aseptate conidia that were ovoid to broadly ellipsoidal in shape (Figure 1E).

### 3.2. Morphological Characterization of the Pathogen

**Figure 2:**
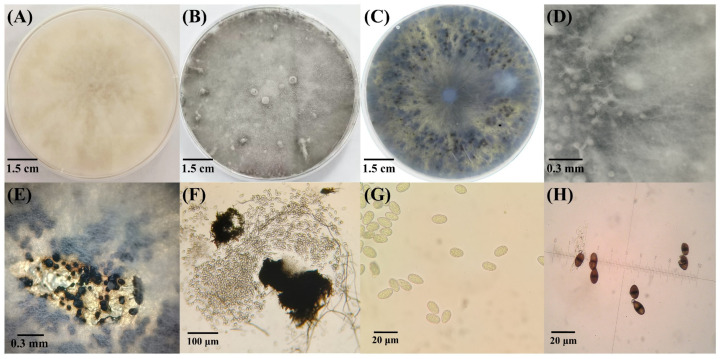
Morphology of a *Lasiodiplodia theobromae* strain on PDA medium. (**A**,**B**): The front of the colony; (**C**): The back of the colony; (**D**–**F**): Pycnidia; (**G**): Immature conidia; (**H**): Mature conidia.

When cultured on PDA at 28 °C in darkness, isolates LYZ0717, LYZ0718, and LYZ0719 initially produced white, floccose colonies and rapidly covered 9 cm-diameter Petri dishes within 3 days (Figure 2A). As incubation progressed, the colonies gradually changed from white to grayish black and eventually to dark brown-black (Figure 2B,C). After 14 days of incubation, black pycnidia developed singly or in clusters on or beneath the colony surface (Figure 2D–F). Immature conidia were hyaline, aseptate, ellipsoidal to broadly ovoid (Figure 2G), and measured 20.86–24.33 × 10.50–13.88 μm. Mature conidia were brown to dark brown, one-septate, and longitudinally striated (Figure 2H), and measured 20.00–28.96 × 11.57–18.56 μm. Collectively, these morphological characteristics were consistent with those of the genus *Lasiodiplodia* [44].

### 3.3. Phylogenetic Analysis

**Figure 3:**
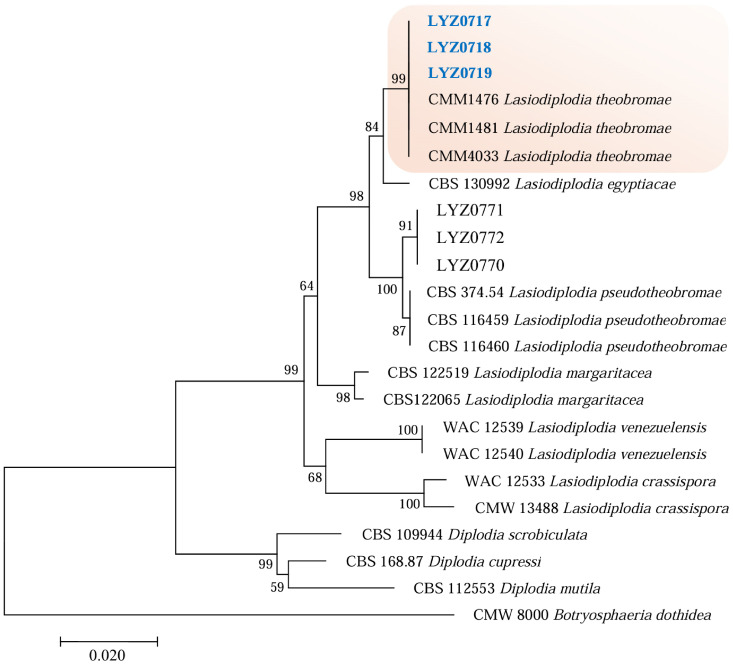
Phylogenetic tree of *L. theobromae* inferred from the combined ITS and *TEF1-α* sequences. Note: Strains LYZ0770, LYZ0771, and LYZ0772 were isolated from stems of pigeonpea plants showing dieback symptoms but were not included as the strains investigated in this study.

A phylogenetic tree was constructed using concatenated sequences of the ITS and *TEF1-α* loci to determine the taxonomic status of the isolates. The phylogenetic tree included the studied isolates LYZ0717, LYZ0718, LYZ0719, LYZ0770, LYZ0771, and LYZ0772, together with representative reference strains of *Lasiodiplodia theobromae*, *L. aegyptiacae*, *L. pseudotheobromae*, *L. margaritacea*, *L. venezuelensis*, and *L. crassispora*, as well as related *Diplodia* species, with *Botryosphaeria dothidea* used as the outgroup taxon. The strains isolated from withered pigeonpea branches, LYZ0717, LYZ0718, and LYZ0719, clustered within a well-supported clade containing multiple reference strains of *L. theobromae*, with a bootstrap support value of 99 (Figure 3). By integrating these molecular data with the morphological characteristics described in Section 3.2, the pathogen responsible for pigeonpea shoot tip dieback in Danzhou City, Hainan Province, was identified as *L. theobromae*.

### 3.4. Pathogenicity of L. theobromae

**Figure 4:**
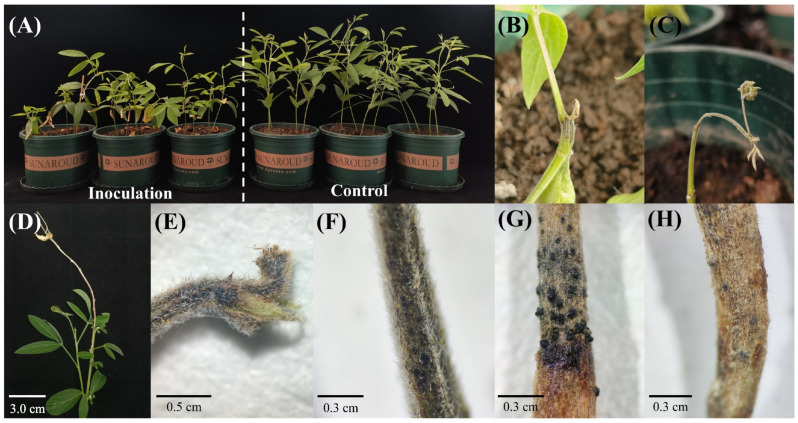
Pathogenicity assay of *L. theobromae*. (**A**): Infected potted plants and control potted plants; (**B**–**D**): Symptoms of spray inoculation with *L. theobromae*; (**E**–**H**): Close-up of stem apex, stem, basal part of stem and root symptoms of diseased plants.

Inoculation assays using spore suspensions of *L. theobromae* reproduced symptoms identical to those observed in the field. The isolates used for inoculation were LYZ0717, LYZ0718, and LYZ0719. Compared to the mock-inoculated controls, the inoculated plants exhibited significantly stunted growth (Figure 4A). The incubation period was determined to be 6 days. Therefore, the first visible symptoms appeared at 6 days post-inoculation. Initial symptoms manifested as dark brown discoloration, softening, and drooping of the shoot tips (Figure 4B). As the infection progressed to the mid-stage, young leaves abscised, and tissue necrosis intensified, extending basipetally (downwards) along the stem (Figure 4C). In advanced stages, the extensive necrosis of the apical shoot induced the proliferation of lateral shoots from the lower stem (Figure 4D). Severely infected plants developed abundant black granular pycnidia on the shoot tips, stems, basal stems, and roots (Figure 4E–H).

### 3.5. Biological Characteristics of L. theobromae

**Figure 5:**
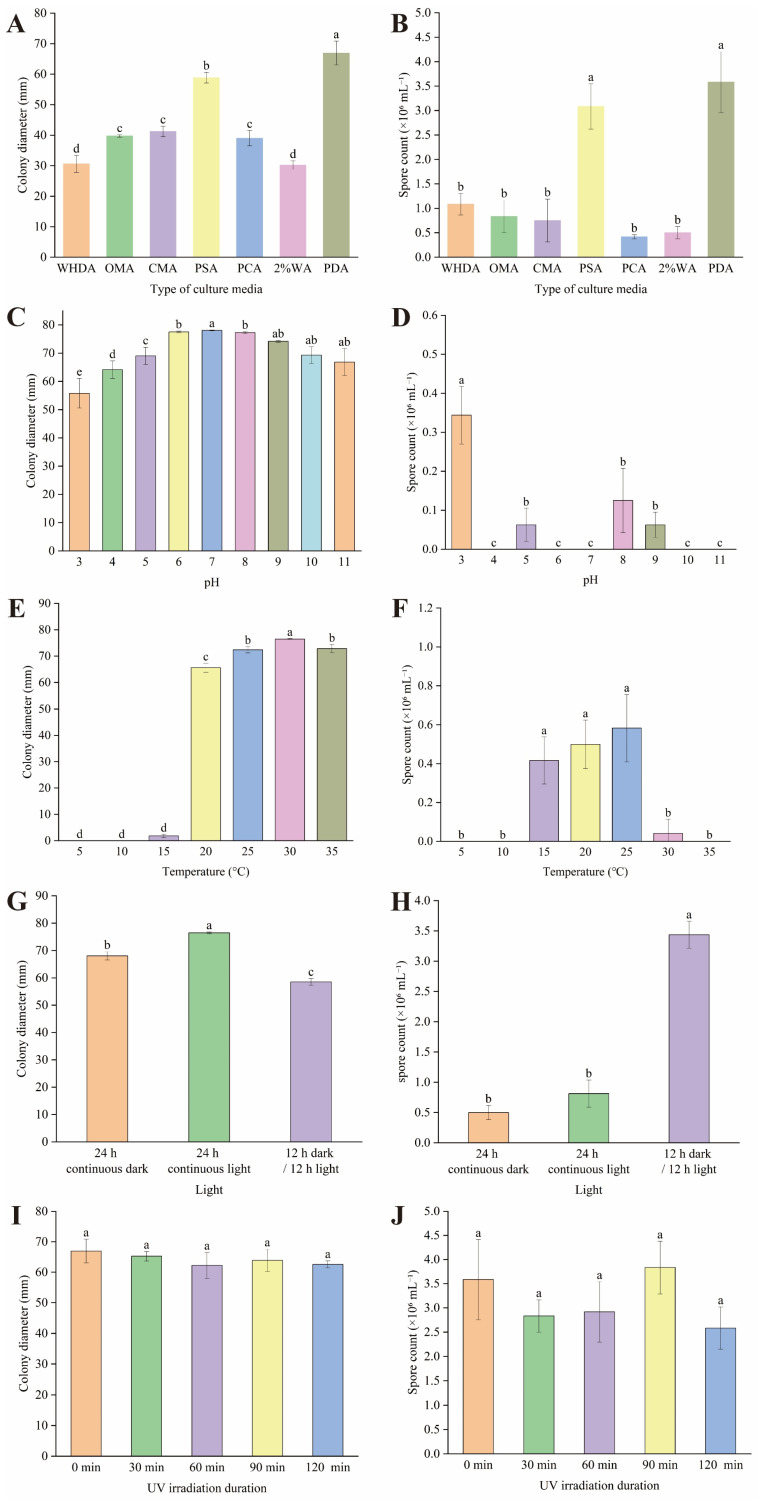
Effects of Different Culture Conditions on Mycelial Growth and Sporulation of *L. theobromae* Isolates LYZ0717, LYZ0718, and LYZ0719. (**A**–**D**,**I**): Mycelial growth of *L. theobromae* under different medium types, pH levels, temperatures, photoperiods, and UV irradiation durations, respectively; (**E**–**H**,**J**): Sporulation of *L. theobromae* under different medium types, pH levels, temperatures, photoperiods, and UV irradiation durations, respectively. Different lowercase letters above the bars indicate significant differences among treatments according to Duncan’s multiple range test at *p* < 0.05.

With the exception of UV irradiation duration, which showed no significant impact on the mycelial growth or sporulation of *L. theobromae*, all other tested culture conditions—including medium type, pH, temperature, and photoperiod—exerted significant effects on both fungal growth and spore production (*p* < 0.05). Among the media tested, PDA was identified as the most suitable for both mycelial growth and sporulation. In contrast, WHDA, OMA, CMA, PCA, and 2% WA media were unfavorable for hyphal expansion and failed to support abundant sporulation (Figure 5A,E). Regarding pH, *L. theobromae* exhibited growth across a broad range from pH 3 to 11. However, the optimal range for mycelial growth was determined to be pH 6–8. Conversely, sporulation was favored by acidic conditions, with maximum spore production observed at pH 3 (Figure 5B,F). In terms of thermal requirements, *L. theobromae* was assessed across a temperature range of 5–35 °C. The optimal temperature for mycelial growth was found to be 25–30 °C. Spore production was negligible at 5–10 °C but reached its peak at 25 °C (Figure 5C,G). Photoperiodic assays revealed distinct requirements for vegetative growth versus reproduction. Continuous light (24 h) was optimal for mycelial growth, whereas an alternating cycle of 12 h darkness/12 h light was most conducive to sporulation. Notably, sporulation levels under this alternating regime were significantly higher than those observed under either continuous light or continuous darkness (Figure 5D,H).

### 3.6. Effects of L. theobromae Isolates LYZ0717, LYZ0718, and LYZ0719 on Pigeonpea Growth

Inoculation with *L. theobromae* significantly compromised the growth and physiological status of pigeonpea plants. All seven evaluated indices—plant height, root length, stem diameter, fresh weight, dry weight, water content, and total flavonoid content—exhibited significant reductions compared to the healthy controls (*p* < 0.05). Specifically, pathogen infection reduced plant height by 45.21%, root length by 38.53%, stem diameter by 29.91%, dry weight by 75.88%, and total flavonoid content by 42.19% (Table 2).

### 3.7. In Vitro Toxicity Assessment of Fungicides

**Figure 6:**
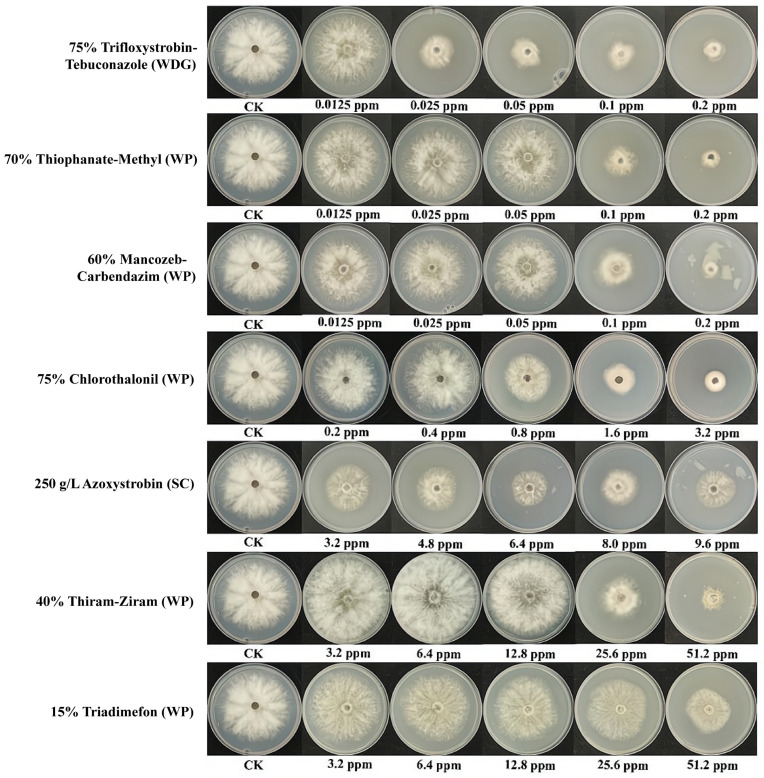
Effects of fungicide-amended plates on the mycelial growth of *L. theobromae* within effective concentration ranges. Note: “CK” stands for normal PDA medium.

Seven commercial fungicides were evaluated for their in vitro inhibitory activity against *L. theobromae*, the causal pathogen of pigeonpea shoot tip dieback. Among the fungicides tested, trifloxystrobin–tebuconazole exhibited the strongest antifungal activity, with the lowest EC_50_ value of 0.0270 ppm, followed by thiophanate-methyl. In contrast, triadimefon showed the weakest inhibitory effect, with an EC_50_ value of 55.9048 ppm. Based on EC_50_ values, the inhibitory activity of the seven fungicides against *L. theobromae* ranked as follows: trifloxystrobin–tebuconazole > thiophanate-methyl > mancozeb–carbendazim > chlorothalonil > azoxystrobin > thiram–ziram > triadimefon (Figure 6; Table 3).

### 3.8. Evaluation of Control Efficacy in Greenhouse Pot Experiments

**Figure 7:**
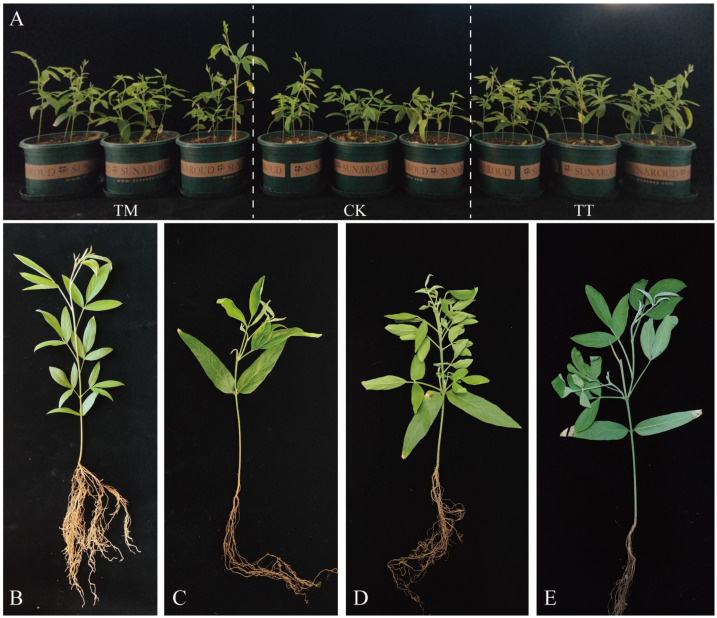
Control efficacy of 70% Thiophanate-Methyl and 75% Trifloxystrobin-tebuconazole against pigeonpea dieback. (**A**): Control efficacy of 70% Thiophanate-Methyl and 75% Trifloxystrobin-tebuconazole against pigeonpea dieback evaluated 10 days post-second application; (**B**): Healthy plants. (**C**): Diseased plants; (**D**): Plants treated with 70% Thiophanate-Methyl; (**E**): Plants treated with 75% Trifloxystrobin-tebuconazole. Note: TM, 70% Thiophanate-Methyl treatment group; CK, untreated control (no fungicide application); TT, 75% Trifloxystrobin-tebuconazole treatment group.

Based on the in vitro fungicide sensitivity results described in Section 3.7, two commercial formulations, 75% trifloxystrobin–tebuconazole and 70% thiophanate-methyl, were selected for greenhouse evaluation against pigeonpea dieback. Disease incidence and disease index were assessed 10 days after the first application and 10 days after the second application. Relative to the inoculated water-treated control (CK), both fungicides reduced symptom development and suppressed disease progression (Figure 7; Table 4). Across the evaluated disease parameters, 70% thiophanate-methyl consistently exhibited greater control efficacy than 75% trifloxystrobin–tebuconazole. These results indicate that both fungicides are effective against *L. theobromae*-induced pigeonpea dieback under greenhouse conditions, with 70% thiophanate-methyl providing superior disease suppression.

## 4. Discussion

*Lasiodiplodia theobromae* is a cosmopolitan, polyphagous fungal pathogen known to colonize or infect more than 500 plant species [14,25,26]. In this study, we provide the first definitive evidence that *L. theobromae* is the causal agent of shoot tip dieback in pigeonpea (*Cajanus cajan*) in China. This finding expands the already extensive host range of this pathogen and identifies pigeonpea as a newly recognized host within legume agroecosystems. Field surveys showed that the disease was widespread in Danzhou, Hainan Province, with incidence ranging from 30% to 100% across fields and pigeonpea cultivars. Diseased plants displayed necrosis of young apical shoots that progressed basipetally, a symptom pattern consistent with *L. theobromae*-associated dieback reported in other host systems [20,23,45,46]. By integrating morphological characterization with phylogenetic analyses of ITS and *TEF1-α* sequences, we achieved reliable species-level identification [17,47]. Together with pathogenicity assays, biological characterization, and fungicide sensitivity tests, these results establish that pigeonpea shoot tip dieback is a disease associated with *L. theobromae* and is reported for the first time in China, providing a basis for targeted disease management and future studies of interactions between pigeonpea and the pathogen.

The morphological evidence obtained in this study provided initial diagnostic support for assigning the representative isolates to *Lasiodiplodia*. The isolates grew rapidly on PDA, initially forming white floccose colonies that later turned gray to dark gray or black, and produced dark pycnidia. Their immature conidia were hyaline, aseptate, and ellipsoidal to broadly ovoid, whereas mature conidia were brown to dark brown, one-septate, and longitudinally striate. These characters are consistent with the diagnostic descriptions of *Lasiodiplodia* and *L. theobromae* [15,18,19]. Nevertheless, morphology alone is not sufficient for reliable species-level identification within Botryosphaeriaceae because closely related species, especially *L. theobromae* and *L. pseudotheobromae*, may show overlapping colony and conidial traits [15,17,19]. In this study, ITS–*TEF1-α* phylogenetic analyses clearly placed isolates LYZ0717–LYZ0719 within the *L. theobromae* clade (Figure 3), and pathogenicity tests demonstrated that these isolates caused typical shoot tip dieback symptoms on *C. cajan* under the tested conditions. Together, the morphological evidence, molecular phylogeny, and pathogenicity assays confirmed *L. theobromae* as the causal agent of pigeonpea shoot tip dieback and underscore the importance of integrating these approaches when diagnosing dieback diseases caused by Botryosphaeriaceae species.

The pathogenic behavior of *L. theobromae* is closely linked to its flexible lifestyle. This fungus can exist as an endophyte, latent pathogen, or active pathogen, depending on host status and environmental conditions [48]. The transition from quiescence to disease development is generally associated with activation of virulence factors and is regulated by genetic, molecular, physiological, and environmental cues [49,50]. Recent studies have begun to reveal potential mechanisms underlying this transition. For example, *L. theobromae* secretes the effector LtCre1, which targets the grape glucose-signaling protein VvRHIP1 and suppresses host immune responses [51]. The pathogen also produces low-molecular-weight phytotoxins that induce necrosis and tissue maceration, thereby facilitating colonization and spread [26]. In addition, multi-omics analyses indicate that environmental factors, particularly temperature, can regulate virulence-associated gene expression. Heat stress may enhance fungal pathogenicity while simultaneously weakening host defense responses, thereby promoting the shift from endophytism to pathogenicity [52,53]. In this context, the warm tropical climate of Hainan may have contributed to the emergence of *L. theobromae* on pigeonpea. The vigorous growth and reproductive capacity observed under warm conditions in this study further support this possibility (Figure 5C,G). These findings also suggest that rising temperatures associated with climate change could increase the risk of host-range expansion and geographic spread of *L. theobromae*, particularly in tropical and subtropical agroecosystems.

The pathogenic effects observed in the present study are consistent with the broad pathogenic potential of *L. theobromae* reported in other pathosystems. In avocado (*Persea americana*), etiological studies have attributed 30–35% of disease incidence to this fungus [14]. In mango (*Mangifera indica*), *L. theobromae* is a major causal agent of gummosis, with reports from India indicating yield losses of 30–100% in severely affected orchards [21]. In grapevine (*Vitis vinifera*), it contributes substantially to Botryosphaeria dieback [49,54], and it has also been associated with twig blight and dieback of blueberry (*Vaccinium corymbosum*) in the Czech Republic and Peru [55,56]. The pathogen has further been implicated in vascular streak dieback of cocoa and stem canker of dragon fruit (*Hylocereus polyrhizus*) in Bangladesh [57,58]. Beyond agronomic crops, its ecological adaptability is reflected by reports from forestry and ornamental hosts, including *Eucalyptus urophylla* × *E. grandis*, *Polyscias balfouriana*, and *Bougainvillea spectabilis* [59]. These reports provide a useful framework for interpreting the severe growth suppression and shoot tip dieback symptoms observed in pigeonpea in the present study. Pathogenicity assays showed that *L. theobromae* substantially suppressed pigeonpea growth, reducing plant height by 45.21% and dry biomass by 75.88% (Table 2). Such pronounced reductions suggest that shoot tip dieback may compromise both pigeonpea productivity and plant vigor, with potentially serious implications in regions where pigeonpea is an important food crop and dietary protein source.

A particularly important finding was that *L. theobromae* infection reduced total flavonoid content in pigeonpea tissues by 42.19% (Table 2). Flavonoids are key secondary metabolites that function as antioxidants and contribute to plant defense, including phytoalexin-associated responses [60,61]. Therefore, the observed decrease may indicate disruption of host secondary metabolism during infection. *Lasiodiplodia* species are known to produce non-host-specific toxins, including lasiodiplodin, that can induce oxidative stress and programmed cell death in host tissues [26]. One plausible explanation is that pathogen-derived pathogenicity-related factors overwhelmed the host oxidative defense system and impaired flavonoid biosynthesis. Alternatively, *L. theobromae* may degrade phenolic compounds enzymatically through ligninolytic enzymes, such as laccases or peroxidases, as reported in related pathogenic processes [62]. Such degradation could facilitate pathogen self-detoxification while weakening host chemical defenses. Although these mechanisms remain to be validated in pigeonpea, the marked reduction in flavonoid content suggests that *L. theobromae* infection affects not only vegetative growth but also biochemical quality traits, thereby broadening the potential economic impact of the disease.

Given the damaging effects of *L. theobromae* on pigeonpea, effective disease management strategies are urgently needed. Fungicide screening remains an important preliminary step for identifying candidate compounds for the management of fungal plant diseases, and evaluations may include both synthetic fungicides and alternative natural compounds [63]. In vitro fungicide sensitivity assays identified 75% trifloxystrobin-tebuconazole and 70% thiophanate-methyl as the strongest inhibitors of *L. theobromae* mycelial growth (Figure 6). Greenhouse pot experiments further showed that these compounds significantly reduced disease severity and limited symptom development (Figure 7; Table 4). However, the in vitro and greenhouse results were not fully concordant. Although 75% trifloxystrobin-tebuconazole exhibited the highest intrinsic toxicity in plate assays, its greenhouse control efficacy was slightly lower than that of 70% thiophanate-methyl. Such discrepancies between in vitro toxicity and in vivo efficacy are common in fungicide evaluations and may reflect differences in fungicide uptake, translocation, persistence, and activity within host tissues [64]. The strong in vitro activity of trifloxystrobin-tebuconazole may be partly attributable to the respiratory inhibition activity of trifloxystrobin against germinating spores [65]. By contrast, thiophanate-methyl, a benzimidazole fungicide, is metabolized in plant tissues to carbendazim and exhibits acropetal translocation through the xylem from roots or application sites toward apical tissues [66]. This systemic movement may be particularly important for controlling pigeonpea shoot tip dieback because *L. theobromae* can colonize vascular and stem tissues during dieback development. Effective fungicides must therefore reach internal infection sites within shoots and stems [37,67]. Our results suggest that systemic mobility, in addition to intrinsic antifungal activity, is a key determinant of in vivo disease control. Although trifloxystrobin-tebuconazole possesses systemic, translaminar, or locally systemic activity [68], its movement within lignified or semi-lignified stem tissues may be more limited than that of benzimidazole fungicides. These findings indicate that fungicide selection for vascular or stem-associated diseases should consider both antifungal potency and systemic distribution within host tissues.

Despite these contributions, several limitations should be acknowledged. Greenhouse trials provide controlled and reproducible evidence of fungicide efficacy, but they may not fully capture the environmental variability, inoculum pressure, plant developmental heterogeneity, and host–pathogen interactions that occur under field conditions. Therefore, multi-location field trials are needed to validate the long-term efficacy, application timing, resistance-management value, and economic feasibility of the candidate fungicides identified here. In addition, although this study establishes the causal agent of pigeonpea shoot tip dieback and quantifies its effects on host growth and flavonoid content, the molecular mechanisms underlying *L. theobromae* pathogenicity on pigeonpea remain unresolved. Future studies should characterize the genetic diversity and population structure of *L. theobromae* in pigeonpea-producing regions, identify virulence determinants involved in host colonization, screen or breed resistant pigeonpea cultivars, and evaluate environmentally sustainable management strategies, including biological control and host-induced resistance. Such efforts will be essential for developing integrated management programs against this emerging disease in tropical and subtropical production systems.

## 5. Conclusions

This study establishes *L. theobromae*, represented by isolates LYZ0717, LYZ0718, and LYZ0719, as the causal agent of pigeonpea shoot tip dieback in China and provides an etiological, biological, and applied framework for disease management. By integrating field diagnosis, morphological and multilocus phylogenetic identification, pathogenicity verification, biological characterization, host-impact assessment, and fungicide evaluation, we convert a previously undefined field syndrome into a clearly delineated and actionable disease entity. The demonstrated capacity of *L. theobromae* to thrive under warm, broad-ranging environmental conditions, severely suppress plant growth, reduce total flavonoid accumulation, and respond differentially to systemic fungicides underscores both its agronomic significance and its management complexity. Importantly, thiophanate-methyl and trifloxystrobin-tebuconazole emerge as practical chemical candidates for disease suppression, with thiophanate-methyl showing superior greenhouse performance. Collectively, these findings provide an etiological, biological, and applied framework for confronting an emerging threat to pigeonpea production in tropical and subtropical agroecosystems.

## Figures and Tables

**Table 2:** Effects of *L. theobromae* Isolates LYZ0717, LYZ0718, and LYZ0719 on the Biological and Chemical Traits of Pigeonpea.

Measurement Index	Control	Treatment	Biomass Loss Rate (%)
Plant height (cm)	25.24 ± 5.08 a	13.83 ± 1.53 b	45.21
Root length (cm)	26.99 ± 7.09 a	16.59 ± 3.38 b	38.53
Stem diameter (mm)	2.24 ± 0.36 a	1.57 ± 0.11 b	29.91
Fresh weight (g)	15.87 ± 5.79 a	6.46 ± 1.52 b	59.29
Dry weight (g)	2.57 ± 1.07 a	0.62 ± 0.17 b	75.88
Water content (%)	83.98 ± 1.18 a	58.49 ± 1.20 b	30.35
Total flavonoid content (mg/g)	4.74 ± 0.65 a	2.74 ± 0.34 b	42.19

Values followed by different letters within the same row are significantly different according to Duncan’s multiple range test (*p* < 0.05).

**Table 3:** Inhibitory Effects of Different Fungicides on *L. theobromae*.

Fungicides	Regression Equation	Correlation Coefficient (r)	EC_50_ (ppm)	95% Confidence Intervals
trifloxystrobin-tebuconazole	y = 0.3902x + 5.6120	0.9984	0.0270	0.0251–0.0291
thiophanate-methyl	y = 0.8963x + 6.0861	0.9836	0.0614	0.0499–0.0756
mancozeb-carbendazim	y = 2.5601x + 7.4187	0.9757	0.1136	0.0821–0.1572
chlorothalonil	y = 2.4007x + 4.7282	0.9646	1.2978	0.9256–1.8198
azoxystrobin	y = 0.8223x + 4.3089	0.9261	6.9250	5.7170–8.3884
thiram-ziram	y = 2.5892x + 1.2299	0.9831	28.5801	21.8825–37.3278
triadimefon	y = 0.9561x + 3.3292	0.9703	55.9048	33.9265–92.1211

Note: The numbers in the regression equation are the regression coefficient and regression intercept from left to right.

**Table 4:** Greenhouse Efficacy of Chemical Fungicides against Pigeonpea Shoot Tip Dieback Caused by *L. theobromae* Isolates LYZ0717, LYZ0718, and LYZ0719.

Evaluation Metrics	Fungicides	Before Application	10 Days After the First Application	10 Days After the Second Application
Disease incidence (%)	CK	25.71 ± 8.35 a	85.71 ± 5.36 a	92.86 ± 12.20 a
	70% thiophanate-methyl	25.00 ± 4.43 a	53.57 ± 3.25 b	61.00 ± 6.41 b
	75% trifloxystrobin-tebuconazole	28.57 ± 2.49 a	57.14 ± 3.78 b	60.71 ± 11.67 b
Disease index (%)	CK	8.93 ± 2.09 a	35.71 ± 8.63 a	41.07 ± 6.11 a
	70% thiophanate-methyl	7.14 ± 2.36 a	13.39 ± 4.32 b	13.75 ± 5.10 b
	75% trifloxystrobin-tebuconazole	7.14 ± 1.62 a	17.86 ± 4.15 b	21.75 ± 8.07 b
Mortality rates (%)	CK	0.00 ± 0.00 a	0.00 ± 0.00 a	3.57 ± 2.45 a
	70% thiophanate-methyl	0.00 ± 0.00 a	0.00 ± 0.00 a	0.00 ± 0.00 a
	75% trifloxystrobin-tebuconazole	0.00 ± 0.00 a	0.00 ± 0.00 a	0.00 ± 0.00 a
Control efficacy (%)	CK	/	/	/
	70% thiophanate-methyl	/	62.49 ± 6.08 a	64.35 ± 8.42 a
	75% trifloxystrobin-tebuconazole	/	49.99 ± 5.62 a	47.04 ± 19.64 a

Note: CK, control plants sprayed with sterile water. Values followed by different lowercase letters within the same column for each evaluation metric are significantly different according to Duncan’s multiple range test (*p* < 0.05).

## Data Availability

All the sequences generated in this study were submitted to GenBank.
